# School nurses' experiences of delivering the UK HPV vaccination programme in its first year

**DOI:** 10.1186/1471-2334-11-226

**Published:** 2011-08-24

**Authors:** Shona Hilton, Kate Hunt, Helen Bedford, Mark Petticrew

**Affiliations:** 1 MRC CSO Social and Public Health Sciences Unit, Glasgow, UK; 2 Centre for Epidemiology and Biostatistics, Institute of Child Health, London, UK; 3 London School of Hygiene and Tropical Medicine, London, WC1E 7HT, UK

**Keywords:** HPV vaccination, cervical, cancer, school nurses

## Abstract

**Background:**

In the United Kingdom (UK) in September 2008, school nurses began delivering the HPV immunisation programme for girls aged 12 and 13 years old. This study offers insights from school nurses' perspectives and experiences of delivering this new vaccination programme.

**Methods:**

Thirty in-depth telephone interviews were conducted with school nurses working across the UK between September 2008 and May 2009. This time period covers the first year of the HPV vaccination programme in schools. School nurses were recruited via GP practices, the internet and posters targeted at school nurse practitioners.

**Results:**

All the school nurses spoke of readying themselves for a deluge of phone calls from concerned parents, but found that in fact few parents telephoned to ask for more information or express their concerns about the HPV vaccine. Several school nurses mentioned a lack of planning by policy makers and stated that at its introduction they felt ill prepared. The impact on school nurses' workload was spoken about at length by all the school nurses. They believed that the programme had vastly increased their workload leading them to cut back on their core activities and the time they could dedicate to offering support to vulnerable pupils.

**Conclusion:**

Overall the first year of the implementation of the HPV vaccination programme in the UK has exceeded school nurses' expectations and some of its success may be attributed to the school nurses' commitment to the programme. It is also the case that other factors, including positive newsprint media reporting that accompanied the introduction of the HPV vaccination programme may have played a role. Nevertheless, school nurses also believed that the programme had vastly increased their workload leading them to cut back on their core activities and as such they could no longer dedicate time to offer support to vulnerable pupils. This unintentional aspect of the programme may be worthy of further exploration.

## Background

Human papillomavirus (HPV) is a common sexually transmitted infection. It is estimated that 20% of sexually active girls will contract the virus by the age of 18 years [1] and up to 80% of women will have had an infection by the time they reach 50 years of age [2]. Despite its frequency, only a small proportion of women who develop persistent infection from high risk genotypes go on to develop cervical cancer; 70% of infections clear within one year and around 90% within two years [3].

The new HPV vaccine was introduced into the United Kingdom (UK) in September 2008, to protect girls against two high risk strains of HPV that cause around 70% of cases of cervical cancer [4]. For the HPV vaccination programme to be most effective it is recommended that young people are given the vaccine before they become sexually active [5]. In view of this, the UK Joint Committee for Vaccination and Immunisation recommended targeting the HPV vaccination programme at girls aged 12 and 13, with a catch-up campaign for girls up to 18 years of age being carried out over a three-year period. To maximise its uptake and effectiveness the three-dose schedule is delivered in schools by school nurses over six months as part of the publicly funded National Health Service. Research has yet to determine whether booster vaccines will be needed at a later date for women who received the vaccine as adolescents, and so continued cervical screening is essential for all women [6]. School nurses play a key role in the successful implementation of the HPV vaccination programme. A key challenge of implementing it will be its acceptability to parents, their daughters and the health care providers (school nurses) involved in delivering the programme in schools. One potential barrier to uptake is a lack of awareness about the role HPV infection plays in cervical cancer [7]. In a recent study of a representative sample of British women (n = 1620), only 2.5% cited HPV as a cause of cervical cancer and awareness of HPV was lowest in those with least formal education [8]. Similarly, in a questionnaire survey of 1032 women attending a Well Woman clinic in London (UK) about 30% recognised HPV only in name. On further questioning, less than half knew of the link with cervical cancer and there was confusion about whether condoms or oral contraceptives could prevent HPV infection [9]. In a more recent study of a representative sample of British women (n = 1620), a quarter of respondents were aware of HPV, and awareness was lower among those with less formal education [8]. Research examining HPV awareness and understandings among the target age of 12 to 18 year old girls has received far less attention despite the acknowledgement that: "Engaging adolescent girls is critical to avoid misconceptions about the protection afforded by HPV vaccination and the future need for cervical screening" [10]. Similarly, the knowledge and attitudes of service providers are known to be important factors in influencing vaccine acceptability, but as yet little is known about school nurses' views on the HPV programme. One qualitative study conducted ahead of the introduction of the programme sought the views of school nurses on vaccinating girls who did not have parental consent. Using in-depth interviews Stretch and colleagues [11] found that school nurses (n = 15) were reluctant to vaccinate if parents had refused permission, felt confused about the legal guidelines governing consent and so would defer vaccination, rather than vaccinate without parental consent. They suggested that to facilitate the legal right of girls below the age of 16 years to self-consent, school nurses would need more training and support on this issue.

In the first year of the programme, HPV vaccine uptake among the 12 to 13 cohort in England has been above 80% and in Scotland almost 90% for the three doses [12,13]. These uptakes rates are higher than those reported from a pilot study in Manchester in which rates of 70.6% (first dose) and 68.5% (second dose) were achieved [14]. These figures also exceed the level at which the programme is deemed to be financially cost effective [5]. Acceptability rates have also been high (over 80%) in the 'catch up' cohort aged 17 to 18 years for all three doses in Scotland. However, in England only one in three 17-18 year olds accepted all three doses [12,13]. Any sub-optimal uptake in the older cohort of girls could be construed as disappointing given that in the UK there are around 2800 new cases of cervical cancer and approximately 950 deaths each year [15] and given the level of media publicity that coincided with the launch of the HPV programme following reality television star Jade Goody's diagnosis and death from cervical cancer [16]. Such publicity was expected to raise public awareness about the 'severity' of cervical cancer, a factor which is known to be important when deciding about vaccination [17]. This study offers some of the first insights from school nurses' perspectives and experiences in delivering the HPV vaccination programme in its first year. In particular, to investigate school nurses'assessment of the HPV vaccine, their experiences of delivering the school based programme, and their views on parental decision-making about HPV vaccination which may help guide its future implementation.

## Methods

### Sampling and recruitment

Thirty in-depth telephone interviews were conducted with school nurses between September 2008 and May 2009 to cover the first year of the HPV vaccination programme in schools in the UK. School nurses were recruited across the UK via adverts on the Royal College of Nurses website (n = 22), posters advertising the study at the 2007 Community Practitioners' and Health Visitors' Annual Conference (n = 5) and from snowballing through GP practices (n = 3). Purposive sampling was used to obtain a diverse sample of school nurses in terms of the number of years' experience as a school nurse, age, geographical location and parental status. School nurses were also targeted to include those working in schools in deprived, affluent, rural and urban locations. To identify our purposive sample we administered a short questionnaire over the telephone prior to interview (see additional file 1, appendix 1).

### Data collection and analysis

The interview schedule was developed from the literature and by first conducting several short telephone interviews with immunisation and child health specialists, public health specialists and health policy makers (n = 10) to gather information on the key issues. The interviews were semi-structured using open-ended questions enabling a qualitative approach to gain deeper insights to explore school nurses' views and experiences of these key areas (see additional file 1, appendix 1). Inductive modes of thinking are particularly useful when the aim is to describe, explore, understand, or explain a particular phenomenon. It may consider the 'what', 'why' and 'how' of the phenomenon, but not in terms of 'how many' or 'how frequently' [18] (p. 7). The schedule included broad questions examining their assessment of the HPV vaccine, their experiences of delivering the school based programme, and their views on parental decision-making about HPV vaccination. In addition, the schedule contained more specific probing questions, and the interviews allowed flexibility for nurses to raise issues themselves.

To obtain a diverse sample, prior to conducting the interview all the potential respondents were asked questions about the number of years' experience as a school nurse, age, geographical location, parental status, caseload. At this stage they were posted a consent form to return in a pre-paid envelope and a time and date for the interview were arranged. All the interviews were conducted by SH by telephone from their own home, and lasted approximately 45 minutes. In keeping with MRC policy all the interview tape recordings were kept in a locked drawer and all names removed from transcripts to ensure anonymity. All the interviews were recorded and transcribed verbatim. Each transcript was checked against the audio-recording, then re-read and thematically coded. Following the principles of the constant comparative method and rigorous analysis, [19] which enable systematic comparisons to be made across the large amounts of data, each transcript was repeatedly re-examined and cross-compared to identify common themes. Once all the relevant themes were coded, a coding frame was developed and SH, KH, HB and MP reviewed the data to identify links between themes, dominant discourses and to identify deviant or contradictory cases [20]. To report the data we have used concise quotes; we indicate where these were 'typical' of a broader range of views or were 'atypical', deviating from commonly held viewpoints. For transparency we have identified the question the facilitator asked and have indicated where topics emerged spontaneously from the nurse participants themselves. Ethical approval for the study was obtained from the research ethics committee of the University of Glasgow's Law, Business and Social Sciences Faculty.

## Results

Thirty school nurses took part in telephone interviews. Seventeen were parents themselves, 4 worked with a predominantly deprived caseload, 3 worked with a predominantly affluent caseload and 23 described their caseload as mixed. They were an experienced group of school nurses with a length of service ranging from 5 years up to 36 years.

### Confidence in the HPV vaccine and programme

School nurses were asked about their feelings towards the HPV vaccine and programme. Most of the school nurses were supportive of the programme and considered themselves well placed to implement it. They frequently mentioned their belief that they were implementing a programme that would make a real difference towards future cervical cancer prevention. For instance, one school nurse said that:

"we (school nurses) have stressed to the girls that they're a very fortunate group of young ladies, you know, the first people that will actually have had a vaccine that will help prevent a cancer and for us to be part of that, it's really actually quite something" (SN 18).

Generally school nurses felt confident in the safety of the HPV vaccine and either mentioned that they planned to consent for their own daughters to have it, or stated hypothetically that if they had a daughter they would have no hesitation in consenting. For example, one school nurse said:

"if I had a daughter I would have no problem in giving her it. I feel quite happy and secure that the vaccine is as safe as it can be and has been tested rigorously" (SN15).

However, when asked whether they would be happy to vaccinate a girl under 16 years of age whose parents had refused to consent for the vaccine, almost all of the school nurses did not think that they would feel comfortable going against the wishes of the parents. No school nurses had actually had to deal with this situation.

Commonly, school nurses mentioned that they felt they were making a positive contribution to girls' health beyond the school years. For example, one school nurse stated: "I'm so pro this vaccine it's not true because, obviously, it's the first one we've got against cancer and we can help protect these girls long-term" (SN26). Similarly, another school nurse stated: "I don't have any qualms about giving the girls the vaccine because I think it's a very positive thing for their future health" (SN28). Whilst most school nurses were extremely positive about HPV vaccination, there were two nurses who were more cautious in talking about the programme and its safety. One questioned the value of the vaccine: "...really how much good the vaccine will do in these kids? In my mind it still needs established" (SN14). The other said:

"I don't know about its safety. I'm just giving it because I've been told to give it. We've been told it's perfectly safe so it probably is, it probably is a good thing, but I don't know" (SN30).

### Feelings about HPV vaccination policy

All the participants were asked a broad question on vaccination policy: 'How do you feel about the decisions the government have made in relation to HPV vaccination policy in the UK?'. Four main issues arose: the age range of the girls; the decision to target girls and exclude boys from the programme; the decision to opt for the Cervarix^**© **^vaccine over its competitor, Gardasil^**©**^; and the perceived policy decision to market the vaccine to prevent cervical cancer rather than to protect against the sexual transmission of HPV infection. Most school nurses mentioned that in their experiences they thought a minority of girls did become sexually active at a very young age and therefore targeting girls as young as 12 years of age seemed "appropriate" and "right" (SN22).

School nurses mentioned that the younger girls were also easier to immunise as parents were more involved in the decision (SN8) and the girls presented with less needle phobia (SN30). However, three school nurses stated that they believed the upper age limit should be extended to take account of the fact that many girls may not have the virus if they have been in longer-term relationships (SN9, SN7, SN30). When asked what they would like the upper age limit to be extended to, answers varied from 21 years to 24 years.

School nurses were divided about whether boys should have been included in the programme. They were all aware of the high cost of the vaccine and this led some to suggest a need to ration this vaccine to girls who have the most to benefit (SN17). It was also suggested that had boys been included in the programme, the uptake rate of the vaccine would be lower because parents would not regard it as relevant for their sons (SN12). One school nurse stated boys would benefit through herd immunity: "obviously if girls are protected against HPV, it'll not be passed on to someone else's son" (SN1). In contrast, there was concern among some school nurses that boys were "being excluded from taking responsibility for health" (SN23), that it "reinforces and promotes the idea that it's a girly thing to look after sexual health" (SN29) and "...it removes any joint responsibility between the sexes for health" (SN16). Some school nurses felt they were "...sending out mixed messages about sexual health responsibility" (SN8).

The decision to opt for the Cervarix^**© **^vaccine over Gardasil^**© **^was widely viewed among school nurse respondents as a cost-based rather than evidence-based decision. Most school nurses stated that before the government announcement to use Cervarix^**© **^they had expected the vaccine of choice to be Gardasil^**© **^on the grounds that "it offers some protection against cervical warts as well as cervical cancer" (SN5). The lack of transparency was spoken about by some school nurses as: "frustrating because people become suspicious and irate when things aren't clear..." (SN17). Despite these concerns most school nurses also mentioned that they thought the decision to opt for Cervarix^**© **^probably made their job of trying to attain a high uptake easier. The primary reason given for this view was because the marketing of the Cervarix^**© **^vaccine had been focused on targeting girls and their parents and so was: "...deliberately very pink and joyful, you know and about doing the right thing for securing their daughter's future health" (SN14) and "...obviously being sold as a girl's cervical cancer prevention jab, not as an unattractive sexually transmitted diseases jab" (SN28). However, this marketing strategy led some school nurses to raise concerns about whether the prime focus on cervical cancer rather than sexually transmitted infections may have missed opportunities to educate parents and teenagers of the need to use barrier methods (SN24) and for girls to attend for future cervical screening (SN21), and some worried that it would make it difficult in the future to extend the programme to include boys (SN17, SN7, SN2).

### Experiences of implementing the programme

Out of the thirty school nurses, twenty four described the introduction of the programme as 'rushed,' 'too hurried' or 'too fast' and many of them stated that in the weeks leading up to its introduction they felt ill prepared. One school nurse described feeling that she and her colleagues were: "...kind of groping in the dark" (SN17). Another recalled: "Oh it's been way too rushed and it's caused a lot of unnecessary panic and stress... really we've been thrown in at the deep end" (SN 28). There was general agreement across the interviews that it could have been planned better and had had a huge impact on their workload. For instance, it was stated:

"... there wasn't enough preparation early enough around doing these vaccines...we didn't have training until a fortnight before it happened, it was way into September (i.e. the month that the programme was introduced into schools in Scotland) and we only got one training day. I mean we knew this programme was going to happen, well, from last year" (SN18).

#### Similarly another school nurse stated

"it was rushed, too hurried. It was really difficult, the decision was made for Cervarix^**© **^just before the summer holidays, we were supposed to get the leaflets and stuff over the summer holidays, which anyway was daft, cos we don't work in summer holidays, so we came back to everything to do. It had a huge impact on my workload" (SN11).

Several school nurses mentioned a lack of planning by policy makers: "I don't think the planners realised that three weeks after they told us which vaccine they were using, the school nurses were going off for their six week break" (SN26). The impact on school nurses' workload was spoken about at length by all the school nurses. They all spoke about prioritising their child protection work and having to cut back on core work such as: planned health education sessions, running drop-in services and delays in health assessments. Of most concern to school nurses was the fact that they were no longer able to dedicate time to offering one to one support to vulnerable pupils and that this might adversely affect their relationship with the pupils to whom they offer regular support. One school nurse described the implementation of the programme as:

"...a logistical nightmare... kids are used to knocking on our door and me being here, if you're not here, they feel you're not here for them, then they want you less, that's the relationship that I have. It will take a knock" (SN25).

#### Another school nurse said

"You feel by not being here you are just another adult not being here for them when they need you, and if the school gets a really bad service they end up looking elsewhere for the support. Once you're taken out of the school then you have to re-establish your relationships again" (SN1).

To prevent the breakdown of established services some school nurses spoke of working additional hours or having requested additional staff to help with the vaccination programme. Where extra staff were provided, these school nurses tended to talk more positively about the programme's implementation. One school nurse suggested that: "...if you're adequately staffed, like we are, it can be absolutely superb no doubt about that" (SN15). In contrast, where school nurses had to cut back on services they had established, either because no additional support was offered or because extra resources were late in coming, school nurses were more likely to air frustrations about its implementation. One frustrated school nurse said:

"In theory, we did have extra resources to manage but it never materialised on the first round and we were doing everything so had to cut out our usual work" (SN18).

Another stated: "We are struggling as a team. It has exhausted everybody. The sickness levels have shot up, and it's frustrating, all our other work ceased completely during the time we were vaccinating" (SN17).

It was common for school nurses to suggest that in its first year of implementation school nurses had played a central role in achieving a high uptake among school girls. One school nurse summed up her views on its implementation:

"I think the success of the programme will be demonstrated by the commitment of the school nurses and their willingness to put the perceived needs of the children and families above their own...bottom line is, as a team we've got together and thought 'well this has got to be done... let's just get on and do it' " (SN 8).

### Perspectives on parental vaccine decision-making

All the school nurses spoke of readying themselves for a deluge of phone calls from concerned parents, but found that in fact few parents telephoned to ask for more information or express their concerns about the HPV vaccine: "I expected to be sitting here at my desk absolutely inundated with calls and I was really not" (SN24). Of the telephone calls they received, most were from parents whose daughters were not in the target age groups in the first wave of the programme, but who were eligible for the catch-up programme, wanting to know when their daughter's school year would be called to receive the vaccine. Other common telephone calls were from parents (usually mothers) wanting to know how to get the vaccine for girls older than 18 years of age. School nurses described these early interactions with parents as "extremely positive" (SN 9) and described parents' interest and willingness for HPV vaccination as "...absolutely astounding" (SN 27).

Based on their experience of delivering the first HPV dose all the school nurses believed they would achieve a high uptake. Some nurses had received a few telephone calls from parents whose daughters had allergies or from parents who did not wish their daughters to have the vaccine. Of the parents who did refuse HPV vaccination, school nurses stated that parents tended to write their reasons for refusal on the back of the consent forms. They identified three main reasons for parental refusal: allergies to latex; a belief that their daughter was not sexually active and therefore not at risk from a HPV infection; and concerns about unknown long-term side-effects of the vaccine on their daughter's fertility or that the vaccine could transmit the HPV infection (SN 8). None of the school nurses recalled any parents refusing HPV vaccination on the grounds that it might encourage their daughters to adopt more risky sexual behaviours.

From in-depth discussions with school nurses, nurses identified four patterns of vaccine decision-making behaviours (we have termed these: active acceptors, passive acceptors, passive rejectors and active rejectors). They also spoke of adopting different strategies to deal with these types of decision-making behaviours.

#### Active acceptors

School nurses generally considered that most parents were actively engaged in the process of making a decision about HPV vaccination and most girls knew why they were getting the vaccine. In their experience the vast majority of parents they had spoken to appeared to be interested in and well informed about the HPV programme and believed that their daughter would be better off vaccinated to reduce the risk from cervical cancer. For example, one school nurse stated:

"most parents understand about cervical cancer, they know that this vaccine is to prevent cervical cancer, which is why their daughters are being given this vaccination" (SN 21).

School nurses reported that few parents contacted them asking for more information or with concerns about the vaccine as further evidence that most parents already 'felt' informed and had found the decision-making process trouble free. For instance one school nurse reported that:

"...although in our covering letter it said please contact the school nurse, nobody wanted more information, they all signed the consent and wanted the vaccine" (SN 16).

When parents did phone with concerns (such as their daughter having a needle phobia), the nurses believed these parents needed reassurance rather than more information about the pros and cons of HPV vaccination.

#### Passive acceptors

However, school nurses also considered that there were a large number of parents and school girls who were not particularly well informed but simply trusted the advice to have HPV vaccination. When asked why they thought so many parents and school girls readily accepted HPV vaccination, school nurses responded that people know about and fear cervical cancer even if they do not understand the role HPV plays in its aetiology. One such response was: "Whatever your educational attainment is, you know what cancer is, people don't really need to know the rest" (SN7). Similarly, another said: "People all know someone who has been affected. Cervical cancer is something that does touch a lot of people's lives" (SN18). According to school nurses the fact the HPV vaccine was often called the 'cervical cancer' vaccine would be enough information for some people.

#### Passive rejectors

The issue of apathy amongst parents was raised by most of the school nurses and spoken about at length by those who were working in schools in very deprived areas. One school nurse stated:

"We have a lot of parental apathy, rather than a parent ringing us up and saying: 'Oh, I'm not very happy about this injection,' we don't really get that side of it. It's more they don't make any decision and don't bother signing the form. I'm having girls coming to me and because in this community the parents have no parental control or interest- I mean that's a broad statement, but I'm talking about ninety percent of my caseload, it's really down to the girls at the end of the day, even if she's only twelve or thirteen, to call the shots" (SN7).

Another school nurse described her frustrations with these parents: "they're just not bothered to send it back (consent form) and the girls would arrive saying, 'oh nobody's signed it' ". She went on to state that for these parents: "health isn't a priority. It's just getting by in life sort of thing, you know, so they don't bother returning the form, they lose the form and they don't bother getting another one" (SN 22). It was also common for school nurses to mention that the older cohort of school girls were more challenging to vaccinate because many would not bother turning up for the vaccine. To obtain consent from such parents and girls a few school nurses spoke about phoning up parents and doing home visits to obtain the required signed consent or cornering girls in the school corridors to ask why they had not turned up. One school nurse recalled a recent experience:

"They couldn't be bothered to read the form or fill it in, you know, motivate themselves enough to do it, but they always wanted the child to have it (the vaccine) as long as somebody bothered to knock on their door and say, 'just sign on the dotted line'. It was just the process of the filling the form in was just too much effort" (SN16).

Another example also mentioned by school nurses as evidence of parental apathy was low attendance numbers at parent information sessions. Nine school nurses had run HPV vaccination information sessions for parents, of which five were described as poorly attended. School nurses suggested a need to follow up these parents and girls with reminder consent forms, targeted information and flexible vaccination sessions.

#### Active rejectors

A fourth group of parents identified by the nurses were those who attended organised information sessions to express what was described as an "anti-immunisation stance" (SN23). These parents were described as being "armed with misinformation" (SN2). They were seen as: "...hostile and challenging to deal with" (SN13), and potentially harmful since: "...they often sounded adamant and could influence other parents to refuse vaccination" (SN1). For instance, one nurse described a parent as being "aggressively disruptive" at an evening information session. She recalled that this parent: "had already made her mind up that her daughter was not going to have the vaccine and just came along simply to be controversial and to simply cause trouble" (SN6). Similarly, another nurse described one parent "dominating discussion" at an information evening she had run and said that the session descended into a question and answer exchange on whether children were being used as "guinea pigs" (SN26). In general school nurses considered that they would be least able to change the views of this group of parents and described offering them the least advice (SN30). School nurses also identified girls in the older cohort as rejecting the HPV vaccine. The main reason cited was that they had a needle phobia. These girls were characterised as lacking an understandings of HPV, the rationale for the vaccine's introduction, and the need to prioritise health.

### Experiences of trying to influence parents and school girls

School nurses were asked, which parents or school girls they found they have least influence in assisting them to make a decision about HPV vaccination. Commonly, nurses suggested that those parents who did phone or attend information sessions were those in least need of the information. Conversely those that they felt would benefit the most tended not to phone or show up. For this reason it was common for school nurses to suggest that parent information session would be ineffectual in affecting vaccine decision-making, stating:

"the same faces turn up...parents that have already made their decisions, whereas the hard to reach parents that haven't given it any thought don't turn up. They look to you (school nurse) to rectify any problem and just sort it out getting their child immunised" (SN 2).

However when asked which parents, if any, they had most influence over, school nurses tended to believe they had most influence over parents with the greatest health needs typically from more deprived backgrounds. One school nurse suggested:

"...I think people from slightly poorer backgrounds expect you to do more hands-on things and be able to fix everything... people from slightly poorer backgrounds are often more likely to actually follow the advice you give, if you have that trust you can engage with them and they are more likely to listen and work with you rather than argue with you, whereas the more affluent parents sometimes think they know best..." (SN 29).

Indeed, it was common for school nurses to assess that as a general rule they have more experience of working with less affluent parents; the more affluent parents they did come into contact with were characterised as less typical of parents generally and very challenging individuals. One school nurse concluded:

"...we (school nurses) have a certain level of knowledge, but we don't have the knowledge that these parents that have spent hours researching- you know- they're sort of referring to twelve sources. They'll have done their own literature review on everything and they will just be challenging your knowledge really- not taking in what you're saying" (SN 16).

When encouraged to identify whether there may be tangible ways to influence the uptake of the HPV vaccine, school nurses suggested a need to continue targeting parents who take little interest in their daughter's health with information, consent forms and flexible HPV vaccination appointments and to do likewise for those older girls who initially refused the vaccine because of needle phobias.

## Discussion

An advantage of using home based telephone interviews was that we found that we could recruit school nurses reasonably easily and that they could speak frankly about their views. However it is possible that the interviews were shorter than would have been the case if they had been face to-face interviews. Another limitation of this study is that the school nurses who participated may not be typical and may represent the more engaged members of their profession; nevertheless much of their experience finds resonance with findings from other studies. The school nurses who took part were generally well informed and willing to recommend HPV vaccination to parents and adolescents. However, findings from a online questionnaire which surveyed health professionals found that there was widespread support for the vaccination programme [21]. Similar to findings of Lansley and Bedford (2003) who examined immunisation co-ordinators' views of the Meningitis C campaign when it was introduced in 1999, much of the credit for the high uptake of vaccines should go to the health professionals implementing the programme [22]. School nurses considered themselves well placed to implement the school based programme and viewed the programme as a means of making a positive contribution to girls' health beyond the school years. Whilst they felt positive about the programme, they also believed that the programme had been unnecessarily rushed and had vastly increased their workload leading them to cut back on their core activities. Of particular concern was that they could no longer dedicate time to offer support to vulnerable pupils which they assessed as having a detrimental effect on the nurses' everyday practice and relationship-building with vulnerable pupils. These findings suggest a shift in responsibilities for health and raise questions over which pupils might be unintentionally left behind as a consequence of the vaccination policies and practices.

In relation to HPV policy, school nurses generally understood the rationale for targeting girls aged 12 to 13 years and, contrary to other findings [23], viewed younger adolescents as good candidates for these vaccines and easier to attain consent. However, school nurses raised concerns that by targeting the HPV programme solely at girls, boys were being excluded from taking responsibility for their role in spreading the virus. Such concerns draw parallels with Polzer and Knabe's assertions that the marketing of HPV vaccines directly at women may reinforce assumptions about the female body as the primary vector of sexually transmitted infections (STIs) [24]. Whilst school nurses did not suggest that the vaccine should also be given to boys at present because of its high cost and need to ration health services, the marketing of the vaccine as a girls' vaccine could have implications for the future programme should it be rolled out to include boys if they do not regard the vaccine as relevant for their health. In this respect 'gendering' vaccines can be problematic. Previous research examining parents' views on vaccine preventable diseases found that rubella and mumps vaccines were perceived to be strongly gendered, rubella being commonly seen as an issue for girls and mumps for boys leading some parents to doubt the need for boys to be vaccinated against rubella, and girls against mumps [25].

As suggested by Szarewski [26] the school nurses found the lack of transparency of the Department of Health's decision to opt for Cervarix^**© **^over Gardasil^**© **^frustrating. However, similar to Kane's suggestion [27], school nurses also supported the decision to opt for Cervarix^**© **^and felt it could more easily be promoted to parents as a cervical cancer vaccine rather than a STI vaccine, enabling them to attain higher uptake. However, some school nurses considered there was a need to promote greater awareness about HPV vaccination to avoid misconceptions that barrier methods and cervical screening are no longer needed. This echoes other research [10].

In relation to HPV vaccine decision-making school nurses identified three main reasons for parental refusal: allergies to latex, a belief that their daughter was not sexually active and therefore not at risk from a HPV infection, and concerns about unknown long-term side-effects of the vaccine on their daughters' fertility or that the vaccine could transmit the HPV infection. School nurses also identified needle phobia as a key reason for refusal among the older girls. In contrast to Monk and colleagues' finding that some parents may not give consent to vaccinate if they think their daughters will be given information that might encourage sexual risk taking [28], school nurses in this study did not recall any parents refusing HPV vaccination on the grounds that it might encourage their daughters to adopt more risky sexual behaviours.

School nurses generally considered that most parents were actively engaged in the process of making a decision and supported the vaccination programme, a finding consistent with research piloting the acceptability of the vaccine among parents in Manchester [14]. School nurses also believed that there were also many parents who were 'passive acceptors' who consented for their daughter to have the vaccine without much thought. Another group of parents that school nurses identified and raised concerns about were the large number of parents who did not consent for their daughters to be immunised due to what was described as 'parental apathy'; we have termed such parents 'passive rejectors'. These parents were identified by school nurses as tending to come from deprived communities for whom health may not be the highest priority. School nurses believed that they could have most influence in increasing HPV uptake among these parents. As such any attempts to improve the uptake of vaccination may be worth targeting at this particular group of parents. Such interventions could include mobile immunisation services to vaccinate and gain parental consent. This research indicates that further research with these hard-to-reach groups into identifying what acceptable measures could be implemented to help overcome existing barriers to immunisation may prove fruitful. One group of parents that school nurses considered that they would be unlikely to influence were those who had rejected HPV immunisation. These 'active rejector' parents were viewed as hostile and disruptive during information sessions. Stretch and colleagues have also reported that parents who attended information evenings did not change their view as they had already made a final decision [11].

## Conclusion

To our knowledge this study offers the first insights into school nurses' experiences of implementing the HPV vaccination programme and these may help guide its future implementation. Overall the first year of the implementation of the HPV vaccination programme in the UK has exceeded school nurses' expectations and some of its success may be attributed to the school nurses' commitment to the programme. It is also the case that other factors, including positive newsprint media reporting that accompanied the introduction of the HPV vaccination programme may have played a role [29]. Nevertheless, school nurses also believed that the programme had vastly increased their workload leading them to cut back on their core activities and as such they could no longer dedicate time to offer support to vulnerable pupils. This unintentional aspect of the programme may be worthy of further exploration.

## Competing interests

We have no competing interests. This study was funded by the Medical Research Council, Population Health Science Research Network. HB has been reimbursed in the past (not in the past five years) by several vaccine manufacturers, for attending and speaking at conferences and conducting research.

## Authors' contributions

SH participated in the design, data collection and analysis, and drafted the manuscript. KH, HB and MP participated in the analysis and commented on drafts of the manuscript. All authors approved the final manuscript.

## Pre-publication history

The pre-publication history for this paper can be accessed here:

http://www.biomedcentral.com/1471-2334/11/226/prepub

## Supplementary Material

Additional file 1 **School Nurse Telephone Interview Schedule**. Recruitment and telephone questionnaire. Click here for file
