# Supplementary material for: School nurses' experiences of delivering the UK HPV vaccination programme in its first year
Source: BMC Infect Dis. 2011 Aug 24;11:226. doi: 10.1186/1471-2334-11-226 (PMC3176210; doi:10.1186/1471-2334-11-226)
Supplement: Additional file 1 — School Nurse Telephone Interview Schedule. Recruitment and telephone questionnaire. [file 1471-2334-11-226-S1.DOC]

**Additional file: Appendix 1**

**School Nurse Telephone Interview Schedule**

**Pre Interview Check List**

1. **Have they read the information sheet and returned the consent form?**
2. **Re-state the following:**

-Length of time (up to 1 hr).

-Voluntary nature of participation.

-Recording the telephone interview.

-Confidentiality – (information kept in locked drawer in MRC unit & not disclosed to anyone outside the research team, destroyed safely after 10 years as per MRC policy).

-Anonymity- (all documents and data identifiable only through ID number and any extracts used in presentations or publications given ID numbers to protect identity, using age, number of years service, region of workplace and type of area caseload)

1. **Check if there are any questions or concerns about the study**
2. **Switch to recorder**

| **Section 1: About you** |
| --- |

Name…………………………………………………………………………

|  |
| --- |

Age

How many years have you been practicing as a nurse?

How many years have you been qualified as a school nurse?

Have you ever attended additional training in childhood or adult immunisation or in assessing research evidence?

When…………………………………………………………………………….

Where……………………………………………………………………………..

Run by whom...............................................................................................

Is your caseload mainly with [ ] disadvantaged [ ] advantaged or [ ] mixed families?

Do you have children of your own?

If yes, what ages are they?

What geographical region do you work in?

Can you start by giving me a description of a normal weeks work as a school, the duties you do and priorities you make?

| **Section 2: About the HPV vaccine** |
| --- |

**GENERAL ASSESSMENT OF HPV VACCINE**

What are your feelings towards the HPV vaccination?

Where do you get information about HPV?

Who is the most useful person or organisation that helps you keep updated on HPV?

How effective is the current system at keeping you abreast of the latest research findings on HPV?

What will be your role in the HPV programme?

What training have you had on HPV?

When…………………………………………………………………………….

Where……………………………………………………………………………..

Run by whom……………………………………………………………………………..

Are there any education needs you have that you feel have not been met?

Have you read any medical or nursing journal or magazine articles on HPV?

Which journals?

What do you think or how have you found the uptake of the vaccine?

Who do you think or have found likely to refuse HPV?

What you think of the media coverage?

What do you think of the HPV publicity campaign?

Did you run educational events for HPV?

If any, what have you found are the main objections to this vaccine?

How safe do you feel vaccines are?

Do you have any safety concerns about HPV?

**VACCINE DECSION MAKING AND CONSENT**

How have you found the issue of parental consent and girls consenting?

Have you have any situations where the parents has refused and girl wanting the vaccine – what did you do?

Is the issue of informed consent clear?

Are there any patterns of vaccine decision-making behaviours that you have noticed in the roll of HPV or vaccines more generally? Do these patterns have implications for your practice?

How engaged do you find parents with vaccine decision-making?

**DELIVERING THE PROGRAMME/ POLICY**

What do you think about the age of girls to be getting it?

Do you anticipate any problems with HPV immunisation? Can you describe them?

Can you describe the preparation, implementation and timing of the programme?

What impact will HPV have on your workload?

What will happen to the work you would normally do?

How do you feel about the decisions the government have made in relation to HPV vaccination policy in the UK?

Prompt-The decision to opt for only girls and to opt for the Cervarix**©** vaccine over Gardasil**©**

Is there anything that we have not spoken about today that you feel would be a useful contribution this research or the development of an information source for health professionals

# POST INTERVIEW CHECK

# Tell them will send £30 reimbursement through the post with an envelope with receipt to be sent back once signed

1. Tell what will happen now to data and when findings will be sent to them
2. Thank for time
